# Adaptive Weighted Graph Fusion Incomplete Multi-View Subspace Clustering

**DOI:** 10.3390/s20205755

**Published:** 2020-10-10

**Authors:** Pei Zhang, Siwei Wang, Jingtao Hu, Zhen Cheng, Xifeng Guo, En Zhu, Zhiping Cai

**Affiliations:** School of Computer, National University of Defense Technology, Changsha 410073, China; zhangpei@nudt.edu.cn (P.Z.); wangsiwei13@nudt.edu.cn (S.W.); hujingtao17@nudt.edu.cn (J.H.); chengzhen16@nudt.edu.cn (Z.C.); guoxifeng13@nudt.edu.cn (X.G.); zpcai@nudt.edu.cn (Z.C.)

**Keywords:** multi-feature, incomplete multi-view clustering, subspace learning, graph fusion

## Abstract

With the enormous amount of multi-source data produced by various sensors and feature extraction approaches, multi-view clustering (MVC) has attracted developing research attention and is widely exploited in data analysis. Most of the existing multi-view clustering methods hold on the assumption that all of the views are complete. However, in many real scenarios, multi-view data are often incomplete for many reasons, e.g., hardware failure or incomplete data collection. In this paper, we propose an adaptive weighted graph fusion incomplete multi-view subspace clustering (AWGF-IMSC) method to solve the incomplete multi-view clustering problem. Firstly, to eliminate the noise existing in the original space, we transform complete original data into latent representations which contribute to better graph construction for each view. Then, we incorporate feature extraction and incomplete graph fusion into a unified framework, whereas two processes can negotiate with each other, serving for graph learning tasks. A sparse regularization is imposed on the complete graph to make it more robust to the view-inconsistency. Besides, the importance of different views is automatically learned, further guiding the construction of the complete graph. An effective iterative algorithm is proposed to solve the resulting optimization problem with convergence. Compared with the existing state-of-the-art methods, the experiment results on several real-world datasets demonstrate the effectiveness and advancement of our proposed method.

## 1. Introduction

Traditional clustering methods [1,2,3,4] usually use a single view to measure the similarity of samples. With the rapid progress of data collection, individual features are not enough to describe data points. Multiple views usually contain supplementary information, which may be beneficial to explore the basic structure of the data. With the development of information technology, data mining and other technologies, many datasets in the real-world can be presented from different perspectives, called multi-view data. For example, the same text can be expressed in various languages. In biometric recognition scope, faces, fingerprints, palm prints and iris could form the different views of multi-view data. In the field of medical diagnosis, different examinations of patients can be regarded as different views. Multi-view data could provide sufficient information than the traditional single feature representation in revealing the underlying clustering structure. Furthermore, distinct views contain specific information of intra-view and complementary information of inter-view, which are negotiated with each other to boost the performance of clustering [5,6,7,8,9,10,11,12,13,14].

Based on different mechanisms, we can divide the existing multi-view clustering methods into four categories. The first category methods refer to multi-kernel clustering. These methods usually combine multiple pre-defined kernels to reach optimal clustering results [12,15,16,17]. The second kind of approach is co-training and co-regularized [18,19]. They iteratively learn multiple clustering results that can provide predicted clustering indices for the unlabeled data from different views. In this way, the clustering results are forced to be consistent across views. The third strategy collaboratively transforms the multi-view information into a compact common binary code space. Then, the clustering process is measured in the Hamming space and enjoys superior algorithm acceleration [20,21]. The last mechanism is the subspace-based multi-view clustering method. It assumes that high-dimensional data points are drawn from various low-dimensional subspaces, and each cluster can be drawn from one of the subspaces [22,23,24,25]. The essential idea is to find several low-dimensional representations embedded in latent spaces and finally attain a united representation for downstream clustering tasks [26]. Besides, aiming at finding a shared low-dimensional latent representation via matrix decomposition, the non-negative matrix factorization (NMF) [27]-based multi-view clustering methods [28,29,30,31] can also be seen as a branch of the subspace-based multi-view clustering method.

Although the algorithms mentioned above have achieved great success in different scenarios, these traditional multi-view clustering algorithms cannot effectively deal with multi-view data with incomplete features. Therefore, the incomplete multi-view clustering algorithms [32,33,34] have attracted extensive attention. To the best of our knowledge, existing incomplete multi-view clustering algorithms can be classified into two categories: non-negative matrix factorization based methods and graph-based methods. The NMF-based methods aim at directly obtaining a common low-dimensional representation through non-negative matrix decomposition. Most of them take the strategy of combining view-specific and common representations into a unified one [35,36,37]. Another representative approach of the NMF-based method is to fill the missing data with average feature values and then use the weighted non-negative matrix factorization to reduce the impact of the missing samples [38]. These NMF-based methods can directly obtain a consistent representation with incomplete samples. However, it is limited to the following two points: (1) when the number of views is more than two, the common parts of views will be significantly reduced and cannot be learned a shared representation between views; (2) NMF-based methods usually neglect the intrinsic structure of data, resulting in an uncompacted representation.

The graph-based incomplete multi-view clustering algorithms are more effective in exploring the geometric structure of data than NMF-based methods. The construction of the graph is essential for the success of clustering. However, it is impossible to construct a complete graph connecting all samples due to the lack of partial samples in incomplete multi-view clustering. To cover this problem, Gao et al. [39] first fill the missing parts and then learn graphs and representations. Zhao et al. [36] utilize NMF to obtain consistent representations to guide the generation of graphs with local structures. However, when the missing rate is high, the filling strategy will dominate the learning of the representation, resulting in the filled samples being connected with each other. Moreover, information fusion refers to fusing multiple sources to achieve consistency. In this stage, multiple views are treated equally, which is unreasonable in real applications.

To address the above issues, we propose a novel incomplete multi-view clustering method, constructing the graphs between instances in the latent embedding subspace. In this manner, we can deal with multi-view data with any number of views. Furthermore, an adaptive weighted mechanism is induced to fuse the graphs with local-structure into a complete graph.

**Figure 1:**
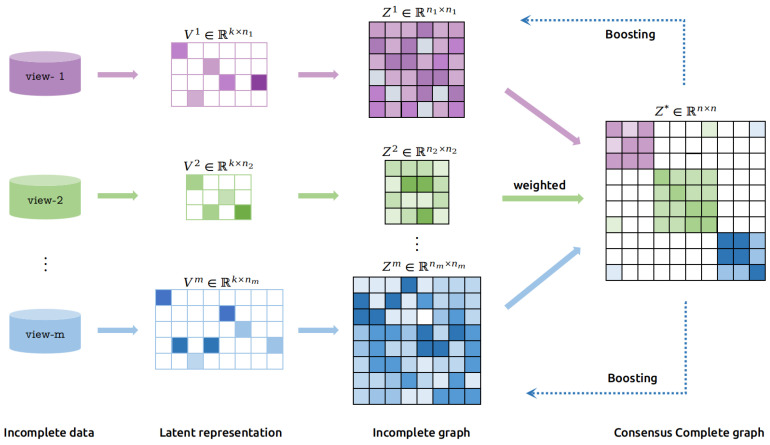
Framework of the proposed adaptive weighted graph fusion incomplete multi-view subspace clustering (AWGF-IMSC). It is a novel incomplete multi-view clustering method to fuse the local-structure contained graph with adaptive view-importance learning. Incomplete graphs of different scales are fused into a complete graph with automatically learning weights. In addition, the constructed complete graph will further guide the learning process of incomplete graphs and latent representations.

In this manner, we establish a relation between missing and unmissing samples. An additional sparse regularization term is imposed on the consensus complete graph to eliminate the adverse effects of inconsistency between views and noise or outliers from each view. Specifically, the framework of this paper is illustrated in Figure 1.

Compared with existing methods, the proposed adaptive weighted graph fusion incomplete multi-view subspace clustering (AWGF-IMSC) algorithm has the following contributions:

- It induces the similarity graph fusion after obtaining latent spaces to extract the local structure of inner views. By virtue of it, noise existing in the original space can be eliminated in latent space and contribute to better graph construction.
- It incorporates relations between missing samples and complete samples into the complete graph. The sparse constraint imposed on the complete graph improves the view-inconsistency and reduces the disagreements between views, making the proposed method more robust in most cases.
- The importance of each view is automatically learned and adaptively optimized during the optimization. Consequently, the important view has strong guidance in the learning process. Moreover, there is no limitation to the number of views in our approach. The proposed method is applicable to any multi-view datasets.

The rest of the paper is organized as follows. The next Section 2 denotes the notations and symbols used in this paper. Section 3 introduces methods mostly related to our work. The proposed algorithm and its optimization process are formulated in Section 4. Besides, we also give the analysis of convergence and complexity of the proposed algorithm in this part. Extensive experiment results and analysis are shown in Section 5, before conclusion and prospectives.

## 2. Notation

For clarity, we give the notation used throughout the paper at the beginning. We use bold letters to represent matrices and vectors. For matrix A, $A_{:,j}$ and $A_{i,j}$ represent its *j*-th column and (i,j) element, respectively. $A^{⊤}$, Tr(A) and $A^{-1}$ denote the transpose, trace and the inverse operations on matrix A, respectively. ${\left\|\cdot \right\|}_{F}$ denotes the Frobenius norm. The $\ell_{2,1}$ norm is denoted as ${\left\|X\right\|}_{2,1}=\sum_{i=1}^{n}\sqrt{\sum_{j=1}^{t}X_{i,j}^{2}}=\sum_{i=1}^{n}{\left\|X_{i,:}\right\|}_{2}$. Moreover, operator $A^{+}$ turns the negative elements in matrix A to 0 while maintaining the non-negative elements, and vice versa. For multi-view datasets $A=\{A^{\left(1\right)},A^{\left(2\right)},\cdots ,A^{\left(m\right)}\}$, the superscript (i) represents the *i*-th view. In individual $A^{\left(i\right)}\in R^{d_{i}\times N}$, each column indicates an instance. *N* is the number of the samples and $d_{i}$ represents the feature dimension of corresponding *i*-th view.

## 3. Related Work

In this section, we will present the work most relevant to the proposed method, i.e., semi-non-negative matrix factorization and subspace learning.

### 3.1. Semi-Non-Negative Matrix Factorization for Single View

Non-negative matrix factorization (NMF) is a significant branch in the field of matrix factorization. NMF aims at finding two non-negative matrix $U\in R_{+}^{d\times K}$ and $V\in R_{+}^{K\times N}$ to roughly approximate the original data matrix, i.e., X≈UV. Since many real-world datasets are usually high-dimensional, the NMF methods have been widely applied in image analysis [40], data mining, speech denoising [41] and population genetics, etc. The semi-NMF [42] is an extension of traditional NMF, which only requires the coefficient matrix to be non-negative. Specifically, given the data matrix $X\in R^{d\times N}$, the semi-NMF utilizes the base matrix $U\in R^{d\times k}$ and the non-negative coefficient matrix $V\in R^{k\times N}$ to approximate the matrix X:

(1) $$\begin{array}{c} \min_{U,V}\|X-UV{\|}_{F}^{2}\;s.t.\;V\geq 0 \end{array}$$.

Ding et al. [42] further propose an iterative optimization algorithm to find the local optimal solution. The updating strategy can be concluded as follows:

With V being fixed, U can be updated by $U=XV^{⊤}{\left(VV^{⊤}\right)}^{-1}.$

With U being fixed, V can be updated by $V_{i,j}\leftarrow V_{i,j}\sqrt{\frac{{\left(X^{⊤}U\right)}_{i,j}^{+}+{\left[V^{⊤}{\left(UU^{⊤}\right)}^{-}\right]}_{i,j}}{{\left(X^{⊤}U\right)}_{i,j}^{-}+{\left[V^{⊤}{\left(U^{⊤}U\right)}^{+}\right]}_{i,j}}}.$

The positive and negative elements of matrix M are denoted as $M_{i,j}^{+}$ and $M_{i,j}^{-}$. And they hold on the property $M_{i,j}=M_{i,j}^{+}-M_{i,j}^{-}$.

NMF and semi-NMF methods are also employed universally in multi-view clustering. Many of the multi-view clustering (MVC) methods utilize NMF to reduce dimension on each view or directly reach a consistent latent presentation [28,43]. Especially in the incomplete multi-view scenario, NMF and semi-NMF play significant roles in achieving a consistent representation from different incomplete views. Li et al. [35] learn a shared representation for the paired instances and view-specific representations for unpaired instances via NMF. The complete latent representation can be attained by combining shared and view-specific representations. The method in [44] utilizes weighted semi-NMF to reach a consensus representation. Then, the $\ell_{2,1}$ norm regularized regression is imposed to align the different basis matrices. Although these NMF-based methods could learn a consensus representation from the incomplete views, the number of views and the absence of local structure limit their performance.

### 3.2. Subspace Clustering

Subspace clustering is an extension of the traditional clustering method which aims at grouping data in different subspaces [45,46]. The self-representation property [47] of subspace clustering aims to represent data points by the linear combinations of themselves. The formulation can be expressed as:

(2) $$\begin{array}{c} \min_{Z}\|X-XZ{\|}_{F}^{2}+\beta R\left(Z\right)\\ s.t.\;0\leq Z_{i,j}\leq 1,Z^{⊤}1=1, \end{array}$$

where $X\in R^{d\times N}$ is the original data, Z is the self-representation coefficient matrix, with each column being a new representation for corresponding data point. β>0 is a trade-off parameter. Since Z reflects the correlations among samples, it can be regarded as a graph and then we can perform spectral clustering algorithm on it to get the final clustering result.

### 3.3. Incomplete Multi-View Spectral Clustering with Adaptive Graph Learning (IMSC-AGL)

In paper [48], a novel graph-based multi-view clustering method is proposed to deal with incomplete multi-view scenarios termed incomplete multi-view spectral clustering with adaptive graph learning (IMSC-AGL). IMSC-AGL optimizes the shared graph from the low-dimensional representations individually formed by each view. Moreover, a nuclear-norm constraint is introduced to ensure the low-rank property of the ideal graph. The mathematical formulation can be written as,

(3) $$\begin{array}{c} \min_{Z^{\left(v\right)},E^{\left(v\right)},F^{\left(v\right)},M}\sum_{v}\left({∥Z^{\left(v\right)}∥}_{*}-\lambda_{1}\mathrm{Tr}\left(F^{\left(v\right)}F^{(v)T}MM^{T}\right)\right)\\ +\sum_{v}\lambda_{2}\mathrm{Tr}\left(F^{(v)T}G^{(v)T}L^{\left(v\right)}G^{\left(v\right)}F^{\left(v\right)}\right)+\sum_{v}\lambda_{3}{∥E^{\left(v\right)}∥}_{1}\\ \;\begin{array}{c} s.t.Y^{\left(v\right)}=Y^{\left(v\right)}Z^{\left(v\right)}+E^{\left(v\right)},Z^{\left(v\right)}1=1,\\ 0\leq Z^{\left(v\right)}\leq 1,Z_{ii}^{\left(v\right)}=0,F^{(v)T}F^{\left(v\right)}=I,M^{T}M=I, \end{array} \end{array}$$

where $Y^{v}$ represents the complete samples in *v*-th view. $Z^{\left(v\right)}$ denotes the respective v-th view’s graph. $F^{\left(v\right)}$ represents the clustering indicator matrix with proper size. Moreover, M refers to the final shared clustering indicator matrix. Although IMSC-AGL achieves considerable performance in various applications, it can still be improved from the number of hyper-parameters and considering to fuse multiple information in a weighted manner.

## 4. Method

### 4.1. Adaptive Weighted Graph Fusion Incomplete Multi-View Subspace Clustering

In this section, we present our adaptive graph fusion incomplete multi-view subspace clustering method (AWGF-IMSC) in detail and give a unified objective function.

For incomplete multi-view data, we remove the incomplete instances and reform as $X^{\left(i\right)}\in R^{d_{i}\times n_{i}}$, where $d_{i}$ and $n_{i}$ represent the feature dimension and the numbers of visible samples of *i*-th view, respectively. We assume that semi-NMF factorizes the input data $X^{\left(i\right)}\in R^{d_{i}\times n_{i}}$ into base matrix $U^{\left(i\right)}\in R^{d_{i}\times k}$ and coefficient matrix $V^{\left(i\right)}\in R^{k\times n_{i}}$. *k* is the dimension of target space and is commonly set to the number of the clusters of $X^{\left(i\right)}$. Considering that the missing samples differ in each view, we learn latent representations of the corresponding visible samples in each view. Therefore, the semi-NMF for individual view can be formulated as:

(4) $$\begin{array}{c} \min_{U^{\left(i\right)},V^{\left(i\right)}}\sum_{i=1}^{m}\|X^{\left(i\right)}-U^{\left(i\right)}V^{\left(i\right)}{\|}_{F}^{2}\;s.t.\;V^{\left(i\right)}\geq 0. \end{array}$$

To further exploit the intra-view similarity structure and the underlying subspace structure, we utilize the self-representation property [47] on the $k\times n_{i}$ dimensional latent representation $V^{\left(i\right)}$ to construct the graph. Thus, we can obtain the different graphs $Z^{\left(i\right)}$ of individual views by solving the following problem:

(5) $$\begin{array}{c} \min_{V^{\left(i\right)},Z^{\left(i\right)}}\sum_{i=1}^{m}\|V^{\left(i\right)}-V^{\left(i\right)}Z^{\left(i\right)}{\|}_{F}^{2}\\ s.t.\;V^{\left(i\right)}\geq 0,0\leq Z_{j,k}^{\left(i\right)}\leq 1,{Z^{\left(i\right)}}^{⊤}1=1, \end{array}$$

where the constraint $0\leq Z_{j,k}^{\left(i\right)}\leq 1$ and ${Z^{\left(i\right)}}^{⊤}1=1$ guarantee a good probabilistic explanation for $Z^{\left(i\right)}$. After obtaining the graphs on each view, the natural idea is to integrate the multiple incomplete information into a complete one. In order to establish the correspondence between the incomplete and complete graphs, we denote the index matrix $O^{\left(i\right)}$. The index matrix $O^{\left(i\right)}\in R^{n_{i}\times N}$ can extract the visible instances of view *i* from the complete graph. To be specific, the matrix $O^{\left(i\right)}$ is defined as:

$$O_{j,k}^{\left(i\right)}=\left\{\begin{array}{cc} 1,\mathrm{if}\;{x_{j}}^{\left(i\right)}\;\mathrm{is}\;\mathrm{the}\;k-\mathrm{th}\;\mathrm{sample}\;\mathrm{in}\;\mathrm{complete}\;\mathrm{dataset}. & \\ 0,\mathrm{otherwise}. \end{array}\right.$$

Through the index matrix, we can achieve the transformation between complete and incomplete graphs: $Z^{\left(i\right)}=O^{\left(i\right)}Z*{O^{\left(i\right)}}^{⊤}$ or ${O^{\left(i\right)}}^{⊤}Z^{\left(i\right)}O^{\left(i\right)}={\hat{Z}}^{*}$. In the second condition, $O^{\left(i\right)}$ expands the graph $Z^{\left(i\right)}$ into ${\hat{Z}}^{*}$, where ${\hat{Z}}^{*}$ has the same size with $Z^{*}$, but the irrelevant items to view *i* are zero.

Owing to the size of the graph and the similarity magnitude differing among views, it is unreasonable to directly add up the multiple graphs. Consequently, we aim to integrate the multiple information into a completed graph with adaptive learning weights ${\left\{\alpha_{i}\right\}}_{i=1}^{m}$. With the help of the index matrix, relevant elements can be extracted from $Z^{*}$. Then, we can adaptively fuse $\{Z^{\left(i\right)}{\}}_{i=1}^{m}$ into a complete graph with auto-learning weights, as illustrated in Equation (6).

(6) $$\begin{array}{c} \min_{\alpha_{i}}\sum_{i=1}^{m}\|\alpha_{i}Z^{\left(i\right)}-O^{\left(i\right)}Z^{*}{O^{\left(i\right)}}^{⊤}{\|}_{F}^{2}\;s.t.\;\alpha_{i}\geq 0,\sum_{i=1}^{m}\alpha_{i}=1, \end{array}$$

where $\alpha_{i}$ is the weight for *i*-th view. It is automatically learned and optimized to illustrate the importance of *i*-th view. In this manner, the complete graph is learned by a weighted combination of incomplete graphs. Besides, with the fusion of beneficial information, the inconsistencies between different views, noise and outliers in individual view are also integrated into the complete graph. Considering that, an additional sparse constraint is added on $Z^{*}$. Therefore, integrating the above parts into an unified objective function, we have our optimization goal as:

(7) minU(i),V(i),αi,Z(i)Z*∑i=1m‖X(i)−U(i)V(i)‖F2+‖V(i)−V(i)Z(i)‖F2+‖αiZ(i)−O(i)Z*O(i)⊤‖F2+λ2‖Z*‖1s.t.V(i)≥0,0≤Zj,k(i)≤1,Z(i)⊤1=1,∑i=1mαi=1,αi≥0,

$\lambda_{1}$ and $\lambda_{2}$ are non-negative trade-off parameters. In the proposed framework, we have four terms: using semi-NMF to obtain latent representation, conducting graph construction with self-representation, adaptive graph fusion and sparse regularizer. Finally, we get a full-size graph $Z^{*}$ incorporating all the sample information in the latent subspace.

### 4.2. Optimization Algorithm for AWGF-IMSC

The constraint problem in Equation (7) is not jointly convex with regard to all the variables. In this section, we propose an alternating iterative algorithm to solve this optimization problem.

#### 4.2.1. Update $U^{\left(i\right)}$

With $V^{\left(i\right)}$, $Z^{\left(i\right)}$, $\alpha_{i}$ and $Z^{*}$ fixed, for each $U^{\left(i\right)}$, we need to solve the following problem,

(8) $$\min_{U}\|X^{\left(i\right)}-U^{\left(i\right)}V^{\left(i\right)}{\|}_{F}^{2}$$

Each of $U^{\left(i\right)}$ can be solved separately since views are independent from each other. Therefore, the optimization problem that we minimize can be rewritten as:

(9) $$\begin{array}{c} L(U^{\left(i\right)})=\mathrm{Tr}\left({X^{\left(i\right)}}^{⊤}X^{\left(i\right)}-2{V^{\left(i\right)}}^{⊤}{U^{\left(i\right)}}^{⊤}X^{\left(i\right)}+{V^{\left(i\right)}}^{⊤}{U^{\left(i\right)}}^{⊤}U^{\left(i\right)}V^{\left(i\right)}\right) \end{array}$$

The solution for $U^{\left(i\right)}$ can be easily obtained by setting the derivation w.r.t. $U^{\left(i\right)}$ to zero.

(10) $$\frac{\partial L(U^{\left(i\right)})}{\partial U^{\left(i\right)}}=-2X^{\left(i\right)}{V^{\left(i\right)}}^{⊤}+2U^{\left(i\right)}V^{\left(i\right)}{V^{\left(i\right)}}^{⊤}$$

Then, we can get the optimal closed-form solution:

(11) $$U^{\left(i\right)}=X^{\left(i\right)}{V^{\left(i\right)}}^{⊤}(V^{\left(i\right)}{V^{\left(i\right)}}^{⊤}{)}^{-1}$$

#### 4.2.2. Update $V^{\left(i\right)}$

Fixing $U^{\left(i\right)}$, $Z^{\left(i\right)}$, $\alpha_{i}$ and $Z^{*}$, the minimum problem for optimizing $V^{\left(i\right)}$ can be simplified as:

(12) $$\begin{array}{c} L(V^{\left(i\right)})=\sum_{i=1}^{m}\|X^{\left(i\right)}-U^{\left(i\right)}V^{\left(i\right)}{\|}_{F}^{2}+\|V^{\left(i\right)}-V^{\left(i\right)}Z^{\left(i\right)}{\|}_{F}^{2}\;s.t.\;V^{\left(i\right)}\geq 0, \end{array}$$

We can update $V^{\left(i\right)}$ in Equation (12) referring to the update strategy in semi-NMF, since the semi-NMF and subspace learning processes are isolated from each other. The partial derivation of $L(V^{\left(i\right)})$ with respect to $V^{\left(i\right)}$ can be obtained as:

(13) $$\begin{array}{cc} \frac{\partial L(V^{\left(i\right)})}{\partial V^{\left(i\right)}}\; & =V^{\left(i\right)}(I-Z^{\left(i\right)}-{Z^{\left(i\right)}}^{⊤}+Z^{\left(i\right)}{Z^{\left(i\right)}}^{⊤})+{U^{\left(i\right)}}^{⊤}U^{\left(i\right)}V^{\left(i\right)}-{U^{\left(i\right)}}^{⊤}X^{\left(i\right)} \end{array}$$

According to the optimization of semi-NMF and the KKT condition, we can get

$$\begin{array}{c} (V^{\left(i\right)}P^{\left(i\right)}+{U^{\left(i\right)}}^{⊤}U^{\left(i\right)}V^{\left(i\right)}-{U^{\left(i\right)}}^{⊤}X^{\left(i\right)}{)}_{j,k}V_{j,k}^{\left(i\right)}=0, \end{array}$$

where $P^{\left(i\right)}=I-Z^{\left(i\right)}-{Z^{\left(i\right)}}^{⊤}+Z^{\left(i\right)}{Z^{\left(i\right)}}^{⊤}$. Note that ${U^{\left(i\right)}}^{⊤}U^{\left(i\right)}=({U^{\left(i\right)}}^{⊤}U^{\left(i\right)}{)}^{+}-({U^{\left(i\right)}}^{⊤}U^{\left(i\right)}{)}^{-}$. Based on this, we can achieve the updating rule for $V^{\left(i\right)}$:

(14) $$\begin{array}{cc} \; & V_{j,k}^{\left(i\right)}\leftarrow V_{j,k}^{\left(i\right)}\sqrt{\frac{{\left(V^{\left(i\right)}(P^{\left(i\right)}{)}^{-}\right)}_{j,k}+[({U^{\left(i\right)}}^{⊤}U^{\left(i\right)}{)}^{-}V^{\left(i\right)}{]}_{j,k}+({U^{\left(i\right)}}^{⊤}X^{\left(i\right)}{)}_{j,k}^{+}}{{\left(V^{\left(i\right)}(P^{\left(i\right)}{)}^{+}\right)}_{j,k}+[({U^{\left(i\right)}}^{⊤}U^{\left(i\right)}{)}^{+}V^{\left(i\right)}{]}_{j,k}+({U^{\left(i\right)}}^{⊤}X^{\left(i\right)}{)}_{j,k}^{-}}.} \end{array}$$

#### 4.2.3. Update $Z^{\left(i\right)}$

When $U^{\left(i\right)}$, $V^{\left(i\right)}$, $\alpha_{i}$ and $Z^{*}$ fixed, the optimization for $Z^{\left(i\right)}$ can be simplified as:

(15) $$\begin{array}{c} \min_{Z^{\left(i\right)}}\sum_{i=1}^{m}\|V^{\left(i\right)}-V^{\left(i\right)}Z^{\left(i\right)}{\|}_{F}^{2}+\lambda_{1}\|\alpha_{i}Z^{\left(i\right)}-O^{\left(i\right)}Z^{*}{O^{\left(i\right)}}^{⊤}{\|}_{F}^{2}\\ s.t.\;0\leq Z_{j,k}^{\left(i\right)}\leq 1,{Z^{\left(i\right)}}^{⊤}1=1. \end{array}$$

Denoting $H^{\left(i\right)}=O^{\left(i\right)}Z^{*}{O^{\left(i\right)}}^{⊤}$, we can obtain the following equivalent question

(16) $$\begin{array}{c} \min_{Z^{\left(i\right)}}\sum_{i=1}^{m}\mathrm{Tr}({Z^{\left(i\right)}}^{⊤}({V^{\left(i\right)}}^{⊤}V^{\left(i\right)}+\lambda_{1}\alpha_{i}^{2}I)Z^{\left(i\right)}-2{Z^{\left(i\right)}}^{⊤}({V^{\left(i\right)}}^{⊤}V^{\left(i\right)}+\lambda_{1}\alpha_{i}H^{\left(i\right)})\\ +{V^{\left(i\right)}}^{⊤}V^{\left(i\right)}+\lambda_{1}{H^{\left(i\right)}}^{⊤}{H^{\left(i\right)}}^{⊤}). \end{array}$$

Setting the derivative with respect to $Z_{:,j}^{\left(i\right)}$ to zero, we can get

$$\begin{array}{c} \frac{\partial L(Z_{:,j}^{\left(i\right)})}{Z_{:,j}^{\left(i\right)}}=\sum_{i=1}^{m}\sum_{j=1}^{n_{i}}Q^{\left(i\right)}Z_{:,j}^{\left(i\right)}+{Q^{\left(i\right)}}^{⊤}Z_{:,j}^{\left(i\right)}-p^{\left(i\right)}=0, \end{array}$$

where $Q^{\left(i\right)}={V^{\left(i\right)}}^{⊤}V^{\left(i\right)}+\lambda_{1}\alpha_{i}^{2}I$ and $p^{\left(i\right)}=2({V^{\left(i\right)}}^{⊤}V^{\left(i\right)}{)}_{:,j}+2\lambda_{1}\alpha_{i}H_{:,j}^{\left(i\right)}$. For each view, we can obtain the following closed-form solution

(17) $$Z_{:,j}^{\left(i\right)}={\left(Q^{\left(i\right)}+{Q^{\left(i\right)}}^{⊤}\right)}^{-1}p^{\left(i\right)}$$

#### 4.2.4. Update $\alpha_{i}$

By fixing $U^{\left(i\right)}$, $V^{\left(i\right)}$, $Z^{\left(i\right)}$ and $Z^{*}$ and removing other terms, the optimization for $\alpha_{i}$ can be transformed into solving Equation (18).

(18) $$\begin{array}{c} \min_{\alpha_{i}}\sum_{i=1}^{m}{\left\|\alpha_{i}Z^{\left(i\right)}-O^{\left(i\right)}Z^{*}{O^{\left(i\right)}}^{⊤}\right\|}_{F}^{2}\;s.t.\;\sum_{i=1}^{m}\alpha_{i}=1,\alpha_{i}\geq 0 \end{array}$$

Note that $H^{\left(i\right)}\alpha_{i}Z^{\left(i\right)}-O^{\left(i\right)}Z^{*}{O^{\left(i\right)}}^{⊤}$. For each view, we can obtain the following Lagrange function:

(19) $$\begin{array}{c} L\left(\alpha_{i}\right)=\alpha_{i}^{2}\mathrm{Tr}({Z^{\left(i\right)}}^{⊤}Z^{\left(i\right)})-2\alpha_{i}\mathrm{Tr}({Z^{\left(i\right)}}^{⊤}H^{\left(i\right)})+(\mathrm{Tr}({H^{\left(i\right)}}^{⊤}H^{\left(i\right)})+\gamma \left(\sum_{i=1}^{m}\alpha_{i}-1\right), \end{array}$$

where γ is the Lagrange multipliers. Setting the derivative of $L(\alpha_{i})$ w.r.t. $\alpha_{i}$ to zero, we can obtain:

(20) $$\begin{array}{c} \alpha_{i}=\frac{2\mathrm{Tr}({Z^{\left(i\right)}}^{⊤}H^{\left(i\right)})-\gamma }{2\mathrm{Tr}({Z^{\left(i\right)}}^{⊤}Z^{\left(i\right)})} \end{array}$$

According to the constraint $\sum_{i=1}^{m}\alpha_{i}=1$, we can compute γ and further get each $\alpha_{i}$.

#### 4.2.5. Update $Z^{*}$

With $U^{\left(i\right)}$, $V^{\left(i\right)}$, $Z^{\left(i\right)}$ and $\alpha_{i}$ fixed, we need to minimize the following objective for $Z^{\left(i\right)}$.

(21) $$\begin{array}{c} \min_{Z^{*}}\sum_{i=1}^{m}\lambda_{1}\|\alpha_{i}Z^{\left(i\right)}-O^{\left(i\right)}Z^{*}{O^{\left(i\right)}}^{⊤}{\|}_{F}^{2}+\lambda_{2}\|Z^{*}{\|}_{1} \end{array}$$

We can get the equivalent element-wise equation:

(22) $$\min_{Z^{*}}\lambda_{1}{\left\|\sum_{i=1}^{r}\alpha_{i}Z_{:,j}^{\left(i\right)}-rZ_{j,q}^{*}\right\|}^{2}+\lambda_{2}\left|Z_{k,q}^{*}\right|$$

Note that j,p differ in different views. *r* is the count of views in which samples *j* and *p* exist simultaneously. If $O_{j,k}^{\left(i\right)}=1$ and $O_{p,q}^{\left(i\right)}=1$, the elements $Z_{k,q}^{*}$ in $Z^{*}$ are composed of the weighted sum of the corresponding instance $Z_{j,p}^{\left(i\right)}$ in view *i*. Specifically, Equation (22) obtains a unique solution:

(23) $$Z_{k,q}^{*}=\left\{\begin{array}{cc} \frac{2\lambda_{1}r\sum_{i=1}^{r}\alpha_{i}Z_{j,p}^{\left(i\right)}-\lambda_{2}}{2\lambda_{1}r^{2}},\; & \;\mathrm{if}\;\sum_{i=1}^{r}\alpha_{i}Z_{j,p}^{\left(i\right)}>\frac{\lambda_{2}}{2\lambda_{1}r}\\ \frac{2\lambda_{1}r\sum_{i=1}^{r}\alpha_{i}Z_{j,p}^{\left(i\right)}+\lambda_{2}}{2\lambda_{1}r^{2}},\; & \;\mathrm{if}\;\sum_{i=1}^{r}\alpha_{i}Z_{j,p}^{\left(i\right)}<\frac{-\lambda_{2}}{2\lambda_{1}r}\\ 0,\; & \;\mathrm{otherwise}\; \end{array}\right.$$

As can be seen in Equation (23), the solution of optimal $Z^{*}$ is a weighted combination of self-representation graph over the view which corresponding samples are visible. Moreover, the noise and outliers will be given a very small value to make the $Z^{*}$ sparse. Therefore, we can get a robust and complete graph revealing all of the relationships of the samples.

### 4.3. Convergence and Computational Complexity

We end up in this section by analyzing the convergence analysis and computational complexity of our proposed method.

Convergence analysis: We first analyze the convergence of the proposed method. Algorithm 1 is a convex problem during the updating of each variable. Each sub-problem obtains a global optimum solution and the value of the objective function is non-increasing until converges. Experiment results in the next section demonstrate this in practice.

Computational complexity analysis: With the optimization process outlined in Algorithm 1, the total time complexity consists of five parts referring to the alternate steps. For incomplete multi-view setting, dimensionality and the number of complete samples varies across different views. With notations in the following, the first stage for computing $U^{\left(i\right)}$ needs $O(d_{i}n_{i}k+n_{i}k^{2}+k^{3}+d_{i}k^{2})$ for the *i*-th view. The time cost of d×n dimensional matrix X multiplying n×k dimensional matrix $V^{⊤}$ is $O(d_{i}n_{i}k)$. Similarly, the time costs of $VV^{⊤}$ and ${\left(VV^{⊤}\right)}^{-1}$ are $O(n_{i}k^{2})$ and $O(k^{3})$, respectively. At last, the result of $XV^{⊤}$ times ${\left(VV^{⊤}\right)}^{-1}$ costs $O(d_{i}k^{2})$. Therefore the total time cost of updating $U^{\left(i\right)}$ is $O(d_{i}n_{i}k+n_{i}k^{2}+k^{3}+d_{i}k^{2})$ for each view. Similarly to updating $U^{\left(i\right)}$, the time cost of updating $V^{\left(i\right)}$ is $O(q\left(kn_{i}^{2}+d_{i}k^{2}+k^{2}n_{i}+kd_{i}n_{i}\right))$, where q is the number of iterations. The time cost of updating $Z^{\left(i\right)}$ is $O(n_{i}\left(n_{i}k^{2}+n_{i}^{3}\right))$. The time cost of updating $\alpha_{i}$ is $O(mn_{i}^{3})$. At last, solving $Z^{*}$ acquires $O(mn_{i}^{2})$. After all, the time complexity of our algorithm is $O(mn_{i}\left(n_{i}k^{2}+n_{i}^{3}+n_{i}+n_{i}k\right))$.
**Algorithm 1:** AWGF-IMSC
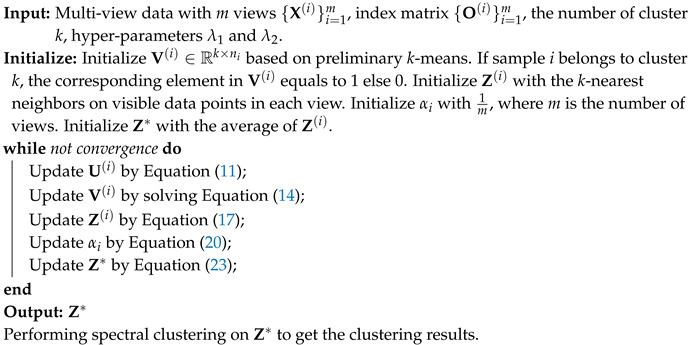


## 5. Experiment

### 5.1. Datasets

To demonstrate the effectiveness of our proposed algorithm AWGF_IMSC, we do comparisons with six baseline methods on four benchmark datasets. The statistical information of the datasets is displayed in Table 1. Detailed introductions are as follows:

- BUAA-visnir face database (BUAA) [49]. The dataset BUAA used in this paper contains 1350 instances of 150 categories. Each instance has visible images (VIS) and near infrared images (NIR), which naturally form a two-view dataset. Both VIS and NIR images are 640×480 pixels. Then, they are resized into 10×10 matrix and vectorized into 100-dimensional features.
- Caltech7 [50]. The Caltech7 dataset is a subset of the Caltech101 dataset, containing seven categories (Face, Motorbikes, Dolla-Bill, Garfield, Snoopy, Stop-Sign and Windsorchair) and 1474 instances. The original images of dataset Caltech7 differ in size. We follow the work in [48], selecting two of five given features as the multi-view dataset. The selected two views refer to 512 dimensional GIST features [51] and 928 dimensional local binary patterns(LBP) features [51].
- One-hundred plant species leaves dataset (100Leaves) [52]. The 100Leaves dataset contains 1600 instances from 100 categories. The original images of 100Leaves differ in size, too. Shape descriptor, fine scale margin and texture histogram features constitute three-views to depict samples from different perspectives.
- Mfeat handwritten digit dataset (Mfeat) [53]. This dataset contains 2000 samples. The size of the original images of dataset Mfeat is 891 × 702 pixels. The public multi-view dataset of it has six views. In our experiments, we select 76-dimensional features of Fourier coefficients of the character shapes and 240-dimensional features of pixel averages.

**Figure 2:**
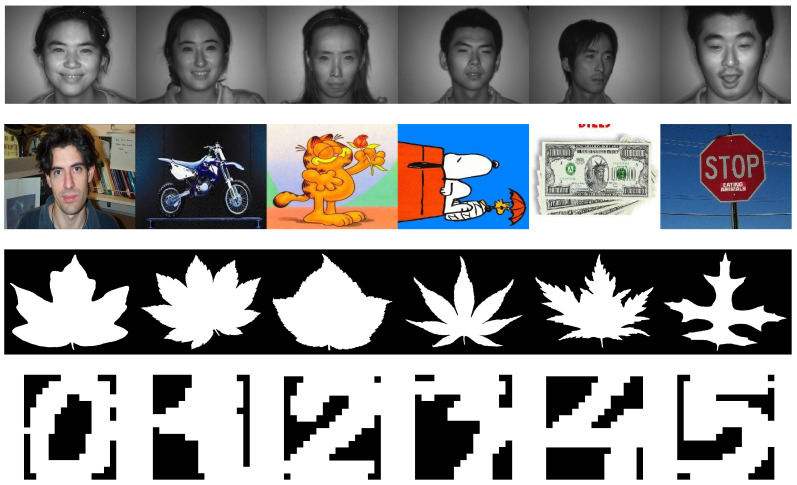
Sample images of the four datasets. Row from top to bottom represents images from BUAA, Caltech7, 100Leaves and Mfeat, respectively.

As shown in Figure 2, we randomly select six pictures of different categories from four original datasets for display.

### 5.2. Baselines

We conduct extensive experiments, comparing with several state-of-the-art incomplete multi-view clustering methods. Brief introductions are given below.

- Best single view (BSV). BSV first fills the missing samples with the average feature values of its view. The affinity matrices can be constructed by Gaussian kernel. Then, we perform spectral clustering algorithm on the similarity matrix of each view and report the best clustering performance.
- Partial multi-view clustering (PVC) [35]. This method supposes that the instances available in both views should have a common representation. The view-specific instances which are missing in another view should maintain the specific information. Based on the NMF, this method integrates the common and view-specific representations in the latent space to form a unified representation.
- Multiple incomplete view clustering via weighted non-negative matrix factorization with $\ell_{2,1}$ regularization (MIC) [38]. This paper first fills the missing instances with an average value of features and then learns a $\ell_{2,1}$ regularized latent subspace by weighted NMF.
- Incomplete multi-modal visual data grouping (IMG) [36]. IMG proposes to use the latent representation to generate a complete graph, which establishes a connection between missing data from different views.
- Doubly aligned incomplete multi-view clustering (DAIMC) [37]. The proposed method first aligns the samples into a common representation by semi-NMF and then aligns the base matrices with the help of $\ell_{2,1}$ regularized regression modal.
- Incomplete multi-view spectral clustering with adaptive graph learning (INMF-AGL) [48] induces a co-regularization term to learn the common representation, which integrates the graph learning and spectral clustering.

For the compared methods, we run their demo with the suggest or default parameters and repeat five times to obtain average results. Note that the PVC and IMG methods can only deal with the two-view scenarios. In our experiments, we combine two different views and report the best result.

Following most existing works, we utilize accuracy (ACC) and normalized mutual information (NMI) to measure the clustering results; higher values representing better clustering performance. Then we give the definition of the two metrics. Denoting true positive (TP), false positive (FP), false negative (FN) and true negative (TN), we can obtain the ACC and NMI definitions as follows:

(24) $$ACC=\frac{TP+TN}{TP+TN+FP+FN},$$

indicating the percentage of correct predicted results in the total samples.

NMI quantifies the amount of information contained in a random variable about another random variable. For clarity, the expression can be formulated as:

(25) $$NMI\left(X,Y\right)=2\frac{I(X,Y)}{H(X)+H(Y)},$$

where mutual information I(X,Y) is $\sum_{x}\sum_{y}p\left(x,y\right)\log\frac{p(x,y)}{p(x)p(y)}$, p(x,y) is the joint probability distribution of *X* and *Y*, p(x) is the marginal probability distribution of *X*. $H\left(X\right)=-\sum_{i}p\left(x_{i}\right)\log p\left(x_{i}\right)$ is the information entropy, regarded as the uncertainty of random variables.

### 5.3. Experiment Setting

In our experiments, we generate incomplete data from complete multi-view datasets in the way of *One-complete*, which means that we randomly select one of the views to be complete. The rest of the views suffer different incomplete ratio (IR) from 10%, 20%, 30%, 40%, 50%, 60%, 70%. For the two-view datasets BUAA, Caltech7 and Mfeat, we randomly select one view as the complete view. The incomplete case occurs in the rest view with randomly removing 10–70% samples. For more than two-view occasions, one view is chosen randomly and the rest views suffer 10–70% missing. The datasets used in this paper can be found in our Github (https://github.com/Jeaninezpp/Incomplete-multi-view-datasets). In our experiments, we perform our proposed method five times as the compared method for fairness. Our code is available at https://github.com/Jeaninezpp/AWGF-code.

### 5.4. Experiment Results and Analysis

Experiment results on different datasets of various compared method are enumerated in Table 2, Table 3, Table 4 and Table 5. These four tables present the ACC results of the above algorithms on four benchmark datasets. Each row shows the accuracy of compared methods under a certain incomplete ratio. We highlight the best results in bold. Each column represents the evolution of the accuracy of the corresponding method as the incomplete ratio increase. Under each incomplete ratio, we can get the sequence number by sorting the accuracy from high to low. The sequence number is regarded as the rank of the algorithms under a certain incomplete ratio. Then, we can obtain the average rank by taking the average of the ranks over each algorithm. The average rank illustrates the robustness of the method in terms of incomplete ratio.

**Figure 3:**
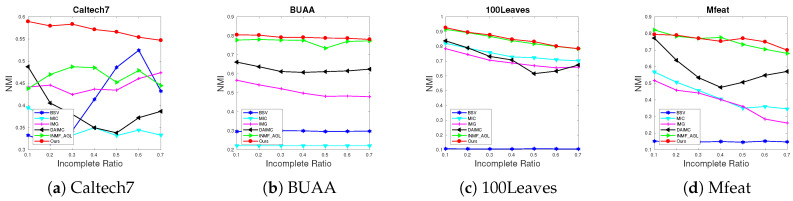
NMI results with different incomplete ratios on four incomplete datasets.

Furthermore, we depict the NMI metric in Figure 3 with line charts. Based on these results, we have the following observations:

- Compared with the proposed method, the BSV method yields worse clustering performance. This is mainly because directly filling the missing instances with the average features will lead them to be clustered into the same group. The weighted NMF methods DAIMC and MIC perform better than BSV at a low missing rate since the NMF-based methods learn a shared representation to exploit the complementary information across views. Besides, the weighted manner reduces the negative impact of the missing instances. However, with the increasing incomplete ratio, these two methods suffer a sharp decline, especially apparent in Mfeat dataset. Methods like IMG and INMF_AGL involving the graph construction perform better than them. Our proposed method integrates the advantages of NMF-based and graph-based methods, adaptively fusing the graph learned from each embedding space. Therefore, our AWGF_IMSC method reaches the best clustering performance in most cases.
- Comparing with the INMF_AGL [48], our proposed method consistently further improves the clustering performance and achieves better results among the benchmark datasets. In addition to the cases on Mfeat, the INMF_AGL method performs higher accuracy than AWGF_IMSC under the 20–50% missing rate. However, our performance exceeds it under the incomplete ratio in 60% and 70%. Although both of INMF_AGL and our AWGF_IMSC adopt subspace clustering to build graph structure in each view, the clustering results demonstrate the effectiveness of graph fusion instead of indicator fusion in INMF_AGL.
- AWGF-IMSC shows clear advantages over other compared baselines under various incomplete ratios, with three best and one second-best results out of the total four datasets. For example, on the BUAA dataset (Table 3), our method transcends the second best method by 7.94%, 7.58%, 7.18%, 7.53%, 14.93%, 9.24% and 7%, respectively. More significant improvements can be seen on dataset Caltech7. In Table 2, the ACCs of the proposed method are 23.42%, 13.10%, 15.02%, 9.74%, 10.48%, 10.8% and 11.43% higher than the second best INMF_AGL method. These significant results verify the effectiveness of the proposed adaptive weighted graph-based fusion learning for incomplete multi-view clustering. Our method achieves the best average rank in Caltech7, BUAA and 100Leaves. In Mfeat, the average rank of the proposed method is second best, which is only 0.43 more than the best, but 0.85 less than the third.
- As shown in Figure 3, we can also observe that the proposed algorithm outperforms other methods on all of the datasets under various incomplete ratio. Besides, our method appears a relatively stable trend as the missing rate increases. Moreover, the abnormal phenomenon of BSV in dataset Caltech7 (Figure 3a) maybe because the preserved complete view has an excellent structure when generating large missing datasets. Therefore, the results of the BSV will be outstanding. Other methods are affected by the negative impact of missing samples and thus produce a lower effect than BSV, while our method is still superior to all compared methods by a more significant proportion, further illustrating the effectiveness and superiority of the proposed method.

### 5.5. Analysis of the Parameter Sensitivity

**Figure 4:**
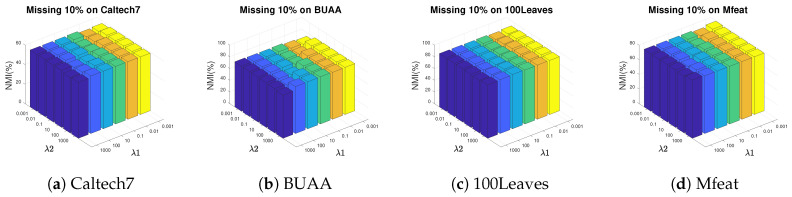
Parameter study on four incomplete datasets. Pictures depict the diversification of NMI under different parameter combinations in the condition of missing 10% instances.

**Figure 5:**
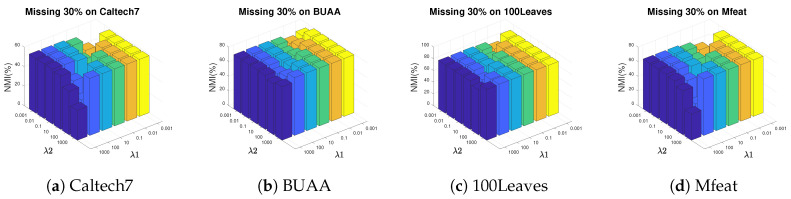
The influence of parameters $\lambda_{1}$ and $\lambda_{2}$ on NMI when 30% instances are missing on four datasets.

In this section, we analyze the impact of the hyper-meters $\lambda_{1}$ and $\lambda_{2}$ in AWGF-IMSC on clustering performance. The parameters are chosen ranging from $\left[10^{-3},10^{-2},\cdots ,10^{3}\right]$ by grid search. Figure 4 and Figure 5 plot the NMI results by varying $\lambda_{1}$ and $\lambda_{2}$ in a large range on BUAA, Caltech7, 100Leaves and Mfeat.

We have the following observations from Figure 4 and Figure 5: (i) All the two hyper-parameters are effective in improving the clustering performance; (ii) AWGF-IMSC is practically stable against these parameters that it achieves competitive performance in a wide range of parameter settings in a low missing rate; (iii) With the incomplete ratio increasing, the combinations of a relatively smaller $\lambda_{1}$ and a more prominent $\lambda_{2}$ tend to achieve better performance. The reason is that the $\lambda_{2}$ controls the impact of the sparse regularizer of the complete graph. Imposing sparseness requirements on the graph within a specific range will filter out the noise in the graph and the inconsistency between the views. (iv) For different datasets, we can conclude that most of the datasets are relatively stable when $\lambda_{1}\in \left\{1e-3,1e-2,1e-1\right\}$, $\lambda_{2}\in \left\{10,100,1000\right\}$.

### 5.6. Convergence Analysis

**Figure 6:**
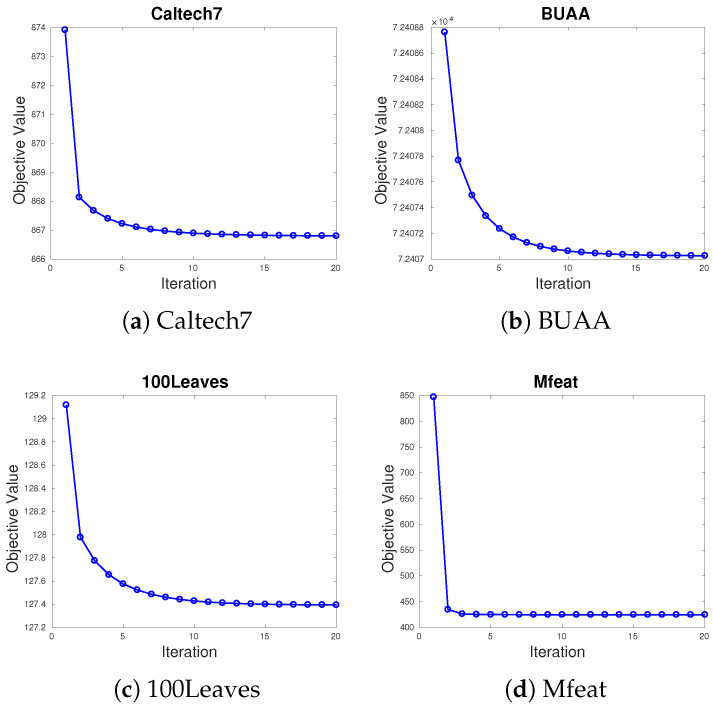
The convergence curves of the objective values on four datasets.

Our algorithm is theoretically guaranteed to converge to a local minimum, as illustrated in the optimization part. For the convergence analysis, we conduct experiments on the four datasets with all incomplete ratios and all suggest parameter scope. We randomly select from each dataset and draw the evolution of the objective value, as shown in Figure 6. In the above experiments, we observe that our algorithm’s objective values monotonically decrease at each iteration and usually converge in less than 20 iterations. These results verify our proposed algorithm’s convergence.

## 6. Conclusions

This article proposes a novel incomplete multi-view clustering method to fuse the local-structure contained graph with adaptive view-importance learning. We incorporate representation learning and incomplete graph fusion into a unified framework, whereas two processes can negotiate with each other, serving for graph learning tasks. A sparse regularization is imposed on the complete graph to do it more robustly to the view-inconsistency. Moreover, the importance of different views is automatically learned, further guiding the construction of the complete graph. We conduct experiments to illustrate the effectiveness and superiority of the proposed method. Some recent works utilize deep neural networks, such as GAN (generative adversarial network), to generate missing features to solve incomplete multi-view clustering problems. The utilization of neural networks to handle multi-view clustering and incomplete multi-view clustering will be advanced considerations in the future.

## Figures and Tables

**Table 1:** Statistics of the datasets.

	Samples	Views	Clusters	Feature		
BUAA	1350	2	150	100	100	
Caltech7	1474	2	7	512	928	
100Leaves	1600	3	100	64	64	64
Mfeat	2000	2	10	76	240	

**Table 2:** ACCs and average ranks on Caltech7 under different incomplete ratios. Bold numbers denote the best result.

IR\Method	BSV	MIC	IMG	DAIMC	INMF_AGL	Ours
10%	0.3328	0.4007	0.5189	0.4105	0.5263	**0.7605**
20%	0.3145	0.3886	0.5027	0.3969	0.5794	**0.7104**
30%	0.3436	0.3205	0.4837	0.4122	0.5791	**0.7293**
40%	0.4139	0.3366	0.5080	0.3423	0.6007	**0.6981**
50%	0.4861	0.3493	0.4290	0.3550	0.5702	**0.6750**
60%	0.5245	0.3446	0.4943	0.4155	0.5874	**0.6954**
70%	0.4324	0.3539	0.4837	0.4109	0.5799	**0.6942**
Average Rank	4.43	5.71	3.29	4.57	2.00	**1.00**

**Table 3:** ACCs and average ranks on BUAA under different incomplete ratios. Bold numbers denote the best result.

IR\Method	BSV	MIC	IMG	DAIMC	INMF_AGL	Ours
10%	0.2964	0.0193	0.3424	0.3203	0.5652	**0.6446**
20%	0.2997	0.0193	0.3424	0.2775	0.5637	**0.6395**
30%	0.3006	0.0193	0.3424	0.2341	0.5566	**0.6284**
40%	0.2997	0.0193	0.3424	0.2336	0.5505	**0.6258**
50%	0.2965	0.0193	0.3424	0.2373	0.4730	**0.6223**
60%	0.2975	0.0193	0.3424	0.2413	0.5293	**0.6217**
70%	0.2979	0.0193	0.3424	0.2596	0.5410	**0.6110**
Average Rank	4.14	6.00	3.00	4.86	2.00	**1.00**

**Table 4:** ACCs and average ranks on 100Leaves under different incomplete ratios. Bold numbers denote the best result.

IR\Method	BSV	MIC	IMG	DAIMC	INMF_AGL	Ours
10%	0.1069	0.6208	0.5661	0.6628	0.8223	**0.8433**
20%	0.1064	0.5768	0.5143	0.5750	0.7835	**0.8104**
30%	0.1060	0.5234	0.4607	0.4740	0.7529	**0.7776**
40%	0.1056	0.4744	0.4302	0.4370	0.7026	**0.7291**
50%	0.1075	0.4665	0.4001	0.3068	0.6551	**0.7028**
60%	0.1065	0.4449	0.3668	0.3405	0.6316	**0.6478**
70%	0.1057	0.4261	0.3740	0.3875	0.5893	**0.6194**
Average Rank	6.00	3.14	4.71	4.14	2.00	**1.00**

**Table 5:** ACCs and average ranks on Mfeat under different incomplete ratios. Bold numbers denote the best result.

IR\Method	BSV	MIC	IMG	DAIMC	INMF_AGL	Ours
10%	0.1520	0.6477	0.5442	**0.8670**	0.8650	0.8100
20%	0.1484	0.5721	0.5004	0.7151	**0.8415**	0.7866
30%	0.1471	0.5220	0.5133	0.5737	**0.8177**	0.7995
40%	0.1497	0.4539	0.4554	0.5042	**0.8148**	0.7864
50%	0.1458	0.3640	0.4037	0.5423	**0.7989**	0.7680
60%	0.1524	0.3583	0.3424	0.5819	0.7214	**0.7515**
70%	0.1476	0.3447	0.3424	0.6446	0.7027	**0.7256**
Average Rank	6.00	4.29	4.71	2.71	**1.43**	1.86

## Abbreviations

The following abbreviations are used in this manuscript:

AWGF-IMSC	Adaptive weighted graph fusion incomplete multi-view subspace clustering
MVC	Multi-view clustering
NMF	Non-negative matrix factorization
BUAA	BUAA-visnir face database
100Leaves	One-hundred plant species leaves dataset
Mfeat	Mfeat handwritten digit dataset
VIS	Visual image
NIR	Near infrared image
LBP	Local binary patterns
IR	Incomplete ratio
ACC	Accuracy
NMI	Normalized mutual information
TP, FP, FN, TN	True positive, false positive, false negative, true negative
BSV	Best single view
PVC	Partial multi-view clustering
MIC	Multiple incomplete view clustering via weighted non-negative matrix factorization with $\ell_{2,1}$ regularization
IMG	Incomplete multi-modal visual data grouping
DAIMC	Doubly aligned incomplete multi-view clustering
INMF-AGL	Incomplete multiview spectral clustering with adaptive graph learning
